# Energy Metabolism and Redox State in Brains of Wistar Audiogenic Rats, a Genetic Model of Epilepsy

**DOI:** 10.3389/fneur.2019.01007

**Published:** 2019-10-01

**Authors:** Carlos Roberto Porto Dechandt, Gustavo Duarte Ferrari, Jonathas Rodrigo dos Santos, José Antonio Cortes de Oliveira, Rui Milton Patrício da Silva-Jr, Alexandra Olimpio Siqueira Cunha, Norberto Garcia-Cairasco, Luciane Carla Alberici

**Affiliations:** ^1^Departamento de Física e Química, Faculdade de Ciências Farmacêuticas de Ribeirão Preto, Universidade de São Paulo, USP, Ribeirâo Preto, Brazil; ^2^Departamento de Fisiologia, Faculdade de Medicina de Ribeirão Preto, Universidade de São Paulo, USP, Ribeirâo Preto, Brazil

**Keywords:** mitochondria, wistar audiogenic rats (WAR), reactive oxygen species, audiogenic kindling, 2,4-dinitrophenol (DNP), N-acetylcysteine (NAC), glycolysis, Na^+^/K^+^ATPase

## Abstract

The Wistar Audiogenic Rat (WAR) strain is a genetic model of epilepsy, specifically brainstem-dependent tonic-clonic seizures, triggered by acute auditory stimulation. Chronic audiogenic seizures (audiogenic kindling) mimic temporal lobe epilepsy, with significant participation of the hippocampus, amygdala, and cortex. The objective of the present study was to characterize the mitochondrial energy metabolism in hippocampus and cortex of WAR and verify its relationship with seizure severity. Hippocampus of WAR naïve (no seizures) presented higher oxygen consumption in respiratory states related to the maximum capacities of phosphorylation and electron transfer system, elevated mitochondrial density, lower GSH/GSSG and catalase activity, and higher protein carbonyl and lactate contents, compared with their Wistar counterparts. Audiogenic kindling had no adding functional effect in WAR, but in Wistar, it induced the same alterations observed in the audiogenic strain. In the cortex, WAR naïve presented elevated mitochondrial density, lower GSH/GSSG and catalase activity, and higher protein carbonyl levels. Chronic acoustic stimulation in Wistar induced the same alterations in cortex and hippocampus. Mainly in the hippocampus, WAR naïve presented elevated mRNA expression of glucose, lactate and excitatory amino acids transporters, several glycolytic enzymes, lactate dehydrogenase, and Na^+^/K^+^ ATPase in neurons and in astrocytes. *In vivo* treatment with mitochondrial uncoupler 2,4-dinitrophenol (DNP) or N-acetylcysteine (NAC) in WAR had no effect on mitochondrial metabolism, but lowered oxidative stress. Unlike DNP, NAC downregulated all enzyme genes involved in glucose and lactate uptake, and metabolism in neurons and astrocytes. Additionally, it was able to reduce brainstem seizure severity in WAR. In conclusion, in WAR naïve animals, both cerebral cortex and hippocampus display elevated mitochondrial density and/or activity associated with oxidative damage, glucose and lactate metabolism pathways upregulation, and increased Na^+^/K^+^ ATPase mRNA expression. Only *in vivo* treatment with NAC was able to reduce seizure severity of kindled WARs, possibly via down regulation of glucose/lactate metabolism. Taken together, our results are a clear contribution to the field of mitochondrial metabolism associated to epileptic seizures.

## Introduction

Epilepsy is one of the most common diseases involving the central nervous system (1). Researches aiming to elucidate pathophysiological mechanisms and allow the development of new therapies to reduce seizure severity are of great value.

Since the 1920s, studies have demonstrated that the ketogenic diet may be antiepileptogenic, suggesting an altered energy metabolism of neurons of patients with epilepsy. Some side effects of the ketogenic diet include certain mitochondrial cytopathies such as Complex I and carnitine deficiencies [for a review (2)], suggesting that mitochondrial energy metabolism plays a central role in the pathology and therapeutics of epilepsy, a complex mechanism yet to be unraveled.

In order to study brain networks participation in the expression of epileptic seizures, animal models of epilepsy have been developed, among them the Wistar audiogenic rat (WAR) strain was developed from inbreeding of Wistar susceptible progenitors (3–5). The WAR strain was selected phenotypically observing their behavioral response to high intensity (117–120 dB) acoustic stimuli. A great variety of alterations in peripheral tissues have been detected in WAR, and some of them are associated with their basal metabolism, such an increase in plasma glucose levels, lactate concentration and adrenergic activity with consequent desensitization of the β-adrenergic lipolytic pathway after acute sound stimulation (6). However, there is no description of mitochondrial energy metabolism in WAR's brains.

The objective of the present study was to characterize the mitochondrial energy metabolism in WAR's hippocampus and cortex, and to verify its relation with seizure severity. We evaluated mitochondrial respiration, redox state, activity of antioxidant enzymes and oxidative stress markers in Wistar and WAR submitted to conditions without seizures (naïve), and after successive epileptic seizures (audiogenic kindling). In addition, we also verified the effects of *in vivo* treatments with a mitochondrial uncoupler or antioxidant in audiogenic kindled WARs.

## Materials and Methods

### Animals

Male Wistar and WAR animals were obtained from the breeding colony at the University of São Paulo, Ribeirão Preto Campus, and the Physiology Department, Ribeirão Preto School of Medicine, respectively. The animals had free access to food and water and maintained on a 12:12 h light:dark cycle at 22°C. All procedures were approved by the Ethics Committee on the Use of Animals of Faculdade de Medicina de Ribeirão Preto—USP (CEUA-FMRP, protocol no. 011/2015).

### Audiogenic Kindling

Wistar and WAR, at approximately 70 days of age, were placed into a cylindrical acrylic arena (height: 32 cm, diameter: 30 cm) inside an acoustically isolated wood chamber (45 × 45 × 40 cm) with a 15-W lamp in the top of the cage. The sound of a ringing bell (120 dB) was digitized with a high-pass filter (N500 Hz) and reproduced with a personal computer coupled to amplifiers and tweeters under the top of the cage. This procedure was repeated twice a day for 10 days. Behavioral evaluations were in accordance with the categorized seizure severity index (cSI) developed by Garcia-Cairasco et al. (7) and modified by Rossetti et al. (8). Sound stimulation lasted 60 seconds or until the animal reached the highest cSI. Twenty-four h after the last (20th) stimulation, animals were euthanized. Cortex and hippocampus were quickly removed. Approximately 5–10 mg were used immediately to measure oxygen uptake, and the remaining were frozen (−80°C) for further analysis.

The term audiogenic kindling was coined by Marescaux et al. (9) in susceptible Wistar rats from Strasbourg, and replicated subsequently in the genetically epilepsy-prone rat (GEPR) strain by Naritoku et al. (10) and in WARs by Garcia-Cairasco et al. (7), Dutra Moraes et al. (11), Romcy-Pereira and Garcia-Cairasco (12). This term refers to the fact that, when submitted to acute acoustic stimulation, those strains develop brainstem-dependent tonic-clonic seizures, and after repeated auditory stimulation, not only change their behavior to a limbic-like phenotype, but based upon their electroencephalogram (EEG), they recruit forebrain structures such as cortex, hippocampus and amygdala. Based upon these protocols, we might say that Wistar rats, which did not present any seizures after repeated stimulations, are not considered “kindled” but “chronically stimulated.” In other words, all the Wistar rats in the chronic protocols of the current study did not display any kind of brainstem or forebrain seizures, and thereafter, will be called “chronically acoustically stimulated” and not “audiogenically kindled,” a term only used for WAR.

### Treatment With N-acetylcysteine (NAC) or 2,4-dinitrophenol (DNP)

WARs with approximately 40 days of age were separated into three groups: WAR, WAR-DNP, and WAR-NAC. The WAR group continuously received drinking water; the WAR-DNP group continuously received drinking water containing 1 mg/L DNP; and the WAR-NAC group continuously received drinking water containing 2 g/L NAC. DNP and NAC solutions were prepared daily and placed into light-protected bottles. Hydric intake was measured daily. Intervention with DNP increased 38% hydric intake while NAC reduced 23%. Relative to body weight, the WAR-DNP group ingested 19.84 ± 1.38 μg/100 g/day DNP, and WAR-NAC group ingested 20.37 ± 0.37 mg/100 g/day NAC, as found in previous studies (13, 14). The DNP dosage used herein is considered 10–100 times lower than those used therapeutically in the past, and over 1,000 times lower than the lethal dose for mice (13, 15, 16). A high dosage of DNP can cause serious health issues, such as cataracts, renal failure and even death (17). DNP is used as a tool to investigate the effects of systemic mild mitochondrial uncoupling on animal energy metabolism and redox state (13). The present NAC dosage is able to increase redox potential by glutathione synthesis (18). A sound stimulus was performed before and after the intervention and cSI was measured. After 40 days, the animals were euthanized, and cortex and hippocampus were quickly removed. Approximately 5–10 mg was used immediately to measure oxygen uptake, and the remainder were stored frozen (−80°C) to the other analysis.

### Permeabilization of Biopsies

Biopsy samples were chopped into 1-mm cubes and placed in ice-cold BIOPS solution [2.7 mM EGTA, 20 mM imidazole, 20 mM taurine, 50 mM acid 2-(N-morfolino) ethanesulfonic potassium, 0.5 mM dithiothreitol, 6.5 mM MgCl_2_, 15 mM phosphocreatine, 0.57 mM ATP, pH 7.1] as recommended by Oroboros Instruments (Innsbruck, Austria). For cell membrane permeabilization, biopsies were placed into BIOPS solution containing saponin (0.01%) during 5 min, at 37°C and 300 rpm stirring. Biopsies were then rinsed with mitochondrial respiration medium MiR05 [0.5 mM EGTA, 3 mM MgCl_2_, 60 mM K-lactobionate, 20 mM taurine, 10 mM KH_2_PO_4_, 20 mM HEPES, 110 mM sucrose, 1 g/L albumin, pH 7.1].

### Respiratory Rates

Respiratory rates were determined monitoring oxygen consumption in an Oxygraph-2k respirometer (Oroboros, Innsbruk, Austria) containing 2.1 mL of air saturated respiration medium. The Respiratory States were determined as follows: NADH-linked, after substrate (9 mM glutamate and 5 mM malate) addition; OXPHOS (phosphorylation), in the presence of adenosine diphosphate (ADP, 1 mM); LEAK (non-phosphorylating), after ATP synthase inhibition by oligomycin (1 μg/mL); ETS (non-coupled), in the presence of the mitochondrial uncoupler carbonyl cyanide m-chlorophenylhydrazone (CCCP, 1 μM); Rox (residual), after complex III inhibition by antimycin A (AA, 3 μM) (19). The value of Rox was subtracted from the other states. After the oxygen consumption measurements, the reaction mixture was completely removed from the oxygraph chamber and submitted to protein quantification using the Bradford method (20).

### Redox State

Reduced and oxidized glutathione were quantified by the fluorimetric ortho-phthalaldehyde method (21). Protein carbonyl was assessed colorimetrically by the selective binding of 2,4-dinitrophenyl hydrazine to protein carbonyl groups (22).

### Citrate Synthase Activity Assay

Citrate synthase activity was measured to estimate the actual value of mitochondrial functional units (23). Approximately 5 mg biopsy was homogenized in 1 mL RIPA buffer [0.75 M NaCl, 0.5% SDS, 0.25 M Tris, 5% Triton X-100, 100 mM EDTA supplemented with 100 mM orthovanadate, 100 mM sodium pyrophosphate, 100 mM PMSF, 1% leupeptin]. Proteins were quantified using Bradford method (20); 2–4 mg of proteins were incubated with reaction medium [50 mM Tris-HCl, 100 μM 5,5′-di-thiobis-(2-nitrobenzoic acid) (DTNB), 0.25% Triton X-100, pH 8.0, supplemented with 50 μM acetyl-CoA] at 37°C for 10 min. The reaction was started with the addition of 250 μM oxaloacetate (24). The results were normalized by protein concentration. Citrate synthase activity was calculated by the following equation:

Citrate synthase activity:

$$\begin{array}{l} \text{Units}\left(\mu \text{mol}/\text{ml}/\text{min}\right)=\frac{\left(\Delta A_{412}\right)/\min\times V\left(mL\right)\times dil}{\varepsilon^{mM}\times L\left(cm\right)\times V_{enz}\left(mL\right)} \end{array}$$

Where V (mL) is the reaction volume, Venz (mL) is the sample volume and εmM is the extinction coefficient of DTNB, which is 13.6 mol/L^−1^.cm^−1^ at 412 nm, L (cm) is the path length for absorbance measurement, which was 0.552 cm for the plates used.

### Catalase (CAT) Activity Assay

CAT activity was measured by monitoring H_2_O_2_ disappearance at 230 nm (25). Approximately 15 mg biopsy was homogenized in 1 mL RIPA buffer at 4°C, centrifuged at 10,000 g for 20 min at 4°C. Supernatant (50 μL) was incubated with 150 μl Tris-EDTA buffer (Tris 1M, EDTA 5 mM) plus hydrogen peroxide (10 mM) for 20 min at 37° C, and absorbance was measured every minute for 5 min. One CAT unit is defined as the amount (mmol) of H_2_O_2_ decomposed in a minute. Results were expressed as U/mg protein.

### Superoxide Dismutase (SOD) Activity Assay

SOD activity was determined by tetrazolium salt using superoxide radicals generated by xanthine oxidase and hypoxanthine monitored at 505 nm (Cayman Chemical, Ann Arbor, MI, USA). Approximately 15 mg biopsy was homogenized in 1 mL RIPA buffer at 4°C, centrifuged at 10,000 g for 20 min at 4°C, 50 μL of supernatant was used for the analysis. One SOD unit is defined as the amount of enzyme needed to promote 50% superoxide radical dismutation. Results were expressed as U/mg protein.

### Lactate Quantification

Lactate concentration was determined using a commercial kit (Labtest Diagnostica SA, Brazil). Approximately 15 mg of sample was homogenized in 1 mL RIPA buffer at 4°C, centrifuged at 10,000 g for 20 min at 4°C and 40 μL supernatant was used for the analysis. Results were expressed as mg lactate/mg protein.

### Analysis of mRNA Expression

Total RNA was isolated using the TRIzol reagent (Invitrogen, Grand Island, NY, USA), solubilized in RNase-free H_2_O, and quantified by measuring the optical density (OD) at 260 nm (NanoDrop spectrophotometer; Thermo Fisher Scientific, USA). For cDNA synthesis, 1.5 μg RNA was used. The mRNA transcript levels were quantified using the Eppendorf Realplex4 Mastercycler Instrument (Eppendorf) and SsOFast EvaGreen (BioRad), according to the manufacturer's instructions. PCR cycling conditions included 10 min at 95°C, followed by 40 cycles at 95°C for 15 s, 60°C for 1 min and 72°C for 60 s. Dissociation curve analysis confirmed that signals corresponded to unique amplicons. After normalization with β-actin, the relative expression of mRNAs was determined by the ΔΔCt method (26, 27). The primer sequences are shown in Table 1.

**Table 1:** Primer sequences used for expression analysis of energy metabolism-related genes.

**Gene**	**Forwad**	**Reverse**
*PGC1α*	CAAGCCAAACCAACAACTTTATCTCT	CACACTTAAGGTTCGCTCAATAGTC
*GS*	GTGCCATAGAGAACGAGCTG	ATCTCGGAAGTAAACCACAGC
*GLUT3*	CTCATCTCCGTTGTCCTCCA	ACCACACCCGCTCCAAT
*GLUT1*	ACCACACTCACCACACTCTG	CCTGCCAAAGCGATTAACAA
*G6PD*	GGAGTTCTTTGCCCGTAA	ATGTTCTTGGTGACTGCTTC
*PFK*	GTGACCAAAGACGTGACCAA	CAACCCGCCCTTAGAGACT
*HK I*	GAACCACGAGAAGAACCAGA	CAGGCAGTCAGCGACAT
*EAAT2*	ATACAACCAAGGCAGTCATC	ACCTAAGACATTCATCCCGT
*EAAT1*	CCAGGCCAACGAAACAC	TGACCCCATTCACAGACC
*PK*	TAAGTCTGGGATGAATGTGG	AGGTCGGTAGAGAATGGGAT
*MCT-3/4*	TCATCACTGGCTTGGGTC	GCACAAAGGAACACGGGA
*MCT-2*	CCTTGGTTACTTCGTCCCC	TGATGTGGCCTGAGACGAG
*MCT-1*	TGGCTATGGCAGGCAG	GACGGACGGTATCGGT
*LDH-5*	GGAATGGCTTGTGCTATC	TCGGAGTCTGGAGAAATAAG
*LDH-1*	CAAACTGCTCATCGTCTCA	ACTCTGCTACTCGTCAAAC
*Na^+^/K^+^ ATPase*	GAGTGCTGGGCTTCAAA	GAGTTTGCCGTAGTAGGGA
*β-actin*	CACTTTCTACAATGAGCTGCG	CTGGATGGCTACGTACATGG

### Transmission Electron Microscopy (TEM)

The animals were perfused through the heart with 0.1M PBS (pH 7.4), followed by 4% paraformaldehyde (PFA) in PBS, pH 7.4. After perfusion, right hippocampi were quickly dissected, washed three times with PBS, and fixed in glutaraldehyde (2.5%) in cacodylate buffer (pH 7.4) for 48 h. Next, tissues were placed in 2% osmium tetroxide (OsO_4_) at room temperature for 2 h, washed with ddH_2_O, dehydrated in a graded series of ethanol (30 to 100%), infiltrated with propylene oxide, embedded in Embed 812 resin (EM Sciences), and polymerized for 72 h at 60°C. Thin sections were stained with uranyl acetate and Reynold's lead citrate for 10 min. Images were recorded on a Jeol JEM- 100 CXII transmission electron microscope. Mitochondrial counts were taken from a series of electron micrographs (20,000x) totaling ~600 μm^2^ for each experimental condition, within each 38.16 μm^2^ field of view. Mitochondrial area (μm^2^) was quantified using NIH-developed Image J software (Wayne Rasband; National Institutes of Health, Bethesda, MD; available at https://imagej.net) and the CLAHE plugin was used to optimize the image contrast. Approximately 16 fields of view were analyzed per condition.

### Data Analysis

Data were expressed as mean ± SEM. One-way analysis of variance followed by Tukey's multiple comparison tests was used to analyze the differences between groups. Differences were considered significant at p < 0.05, using GraphPad Prism®, USA.

## Results

### Effects of Audiogenic Kindling on Seizure Severity Index (cSI)

Wistar and WAR were submitted to chronic stimulation and the seizure behaviors were categorized according to Rossetti et al. (8). The Wistar group did not display any seizure behavior (cSI = 0.0), while the WAR group showed typical seizure behaviors in all stimuli (cSI = 6.8 ± 0.6 and 5.5 ± 0.8 for first and last stimulus, respectively), in agreement with previous studies (28–34).

### Effects of Audiogenic Kindling on Mitochondrial Metabolism and Redox State in Hippocampus

**Figure 1:**
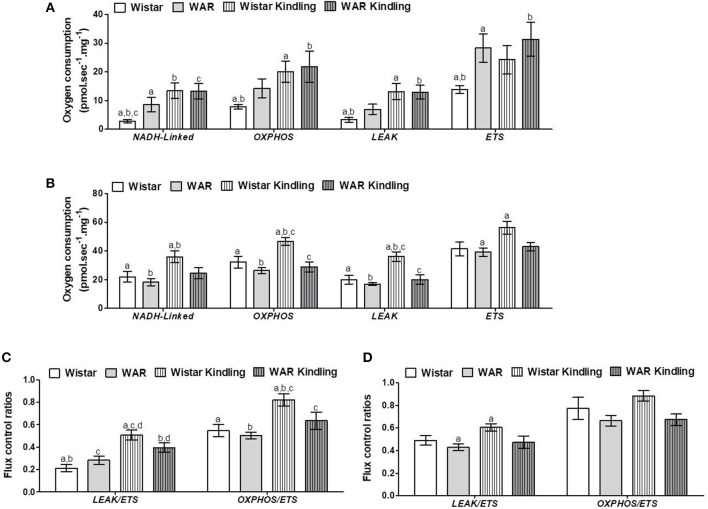
Mitochondrial respiration in brain biopsies from WAR and Wistar rats submitted to chronic stimulation (kindling) or not (naïve). Oxygen consumption in states of NADH-Linked, OXPHOS, LEAK, NON-COUPLED (ETS) in **(A)** hippocampus, or **(B)** cortex. Flux control ratios (FCRs) in **(C)** hippocampus or **(D)** cortex. Mean ± SEM, *N* = 8. Equal letters represent significant differences.

**Figure 2:**
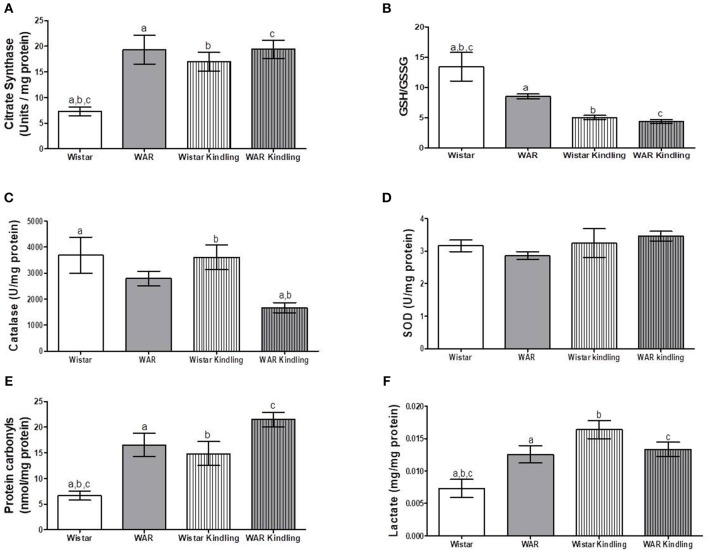
Mitochondrial content and redox state in hippocampus biopsies from WAR and Wistar rats submitted to chronic stimulation (kindling) or not (naïve). **(A)** Citrate synthase content, **(B)** GSH/GSSG ratio, **(C)** Catalase, and **(D)** Superoxide Dismutase (SOD) activities, **(E)** Protein carbonyl content, and **(F)** Lactate content. Mean ± SEM, *N* = 8. Equal letters represent significant differences.

**Figure 3:**
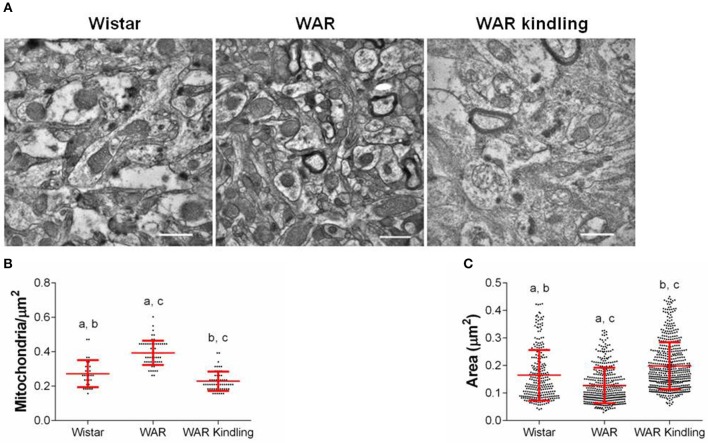
Mitochondrial density and morphology in hippocampus from WAR and Wistar rats (naïve) or WAR submitted to chronic stimulation (kindling). **(A)** Representative electron micrographs, quantifications of mitochondrial **(B)** density, and **(C)** area. Data represent mean of >300 mitochondria (>15 fields of view for 2 rats) ± S.D. for the dot plot graphs.

After hippocampus removal, oxygen consumption and redox state were monitored in the whole tissue (biopsy). Biopsies from WAR group presented higher oxygen consumption compared to Wistar rats in almost all respiratory states: NADH-linked (in the presence of exogenous NADH-linked substrates) and in respiratory states related to the maximum capacities of phosphorylation and electron transfer system, such as OXPHOS (in the presence of exogenous ADP), and non-coupled (ETS, in which the protonophore CCCP collapses the electrochemical H^+^ potential across the inner mitochondrial membrane, stimulating maximal electron transfer system and respiration) (Figure 1A). These high respiration rates in WAR group correlated with an increase in mitochondrial density, evidenced by elevated citrate synthase content (Figure 2A), a classical marker of mitochondrial number (23). Transmission electron microscopy (TEM) confirmed the last result, showing an increased number of mitochondria/area (Figures 3A,B) and showed that WARs have smaller mitochondria than Wistar rats (Figure 3C). Chronic acoustic stimulation in Wistar rats induced alterations observed in naïve WAR, such as high oxygen consumption in the NADH-Linked, OXPHOS, and LEAK states (Figure 1A) and mitochondrial content (Figure 2A). Although changing the TEM parameters (Figure 3C), no significant changes were observed on mitochondrial respiration and citrate synthase activity in WAR submitted to audiogenic kindling compared to naïve WAR (Figure 1A). In addition, calculation of the flux control ratios (FCRs, Figure 1C) also demonstrated increased phosphorylation capacity (OXPHOS/ETS) and uncoupling degree (LEAK/ETS) per mitochondria in Wistar chronically stimulated group compared to naïve Wistar. Biopsies from WAR group also presented higher oxidized state compared to Wistar, as shown by reduced GSH/GSSG (Figure 2B) and catalase activity (Figure 2C), in addition to higher protein carbonyl content (Figure 2E). Additionally, chronic acoustically stimulated Wistar rats also presented an elevation in oxidized state as observed in naïve WAR. No change was found in SOD activity (Figure 2D). Biopsies from the WAR group presented augmented lactate content (Figure 2F), a characteristic observed in several regions of the brain after epileptic seizure (35–37). Lactate content was elevated in Wistar rats submitted to chronic acoustic stimuli; an alteration already observed in naïve WARs (Figure 2F).

### Effects of Audiogenic Kindling on Mitochondrial Metabolism and Redox State in Cortex

**Figure 4:**
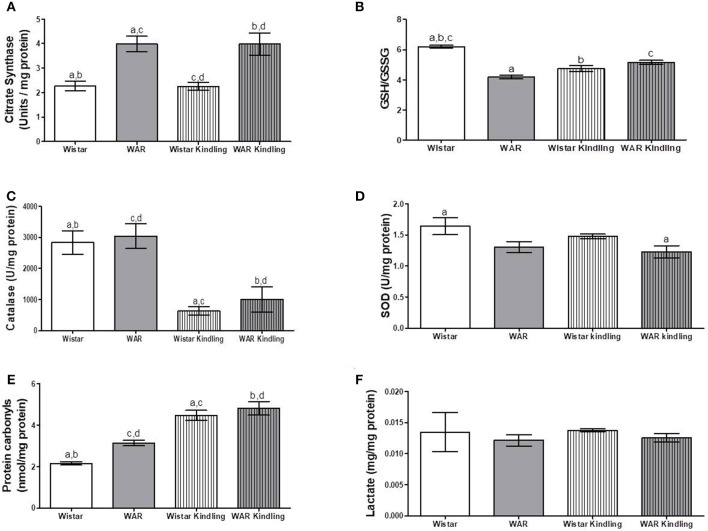
Mitochondrial content and redox state in cortex biopsies from WAR and Wistar rats submitted to chronic stimulation (kindling) or not (naïve). **(A)** Citrate synthase content, **(B)** GSH/GSSG ratio, **(C)** Catalase, and **(D)** Superoxide Dismutase (SOD) activities, **(E)** Protein carbonyl content, and **(F)** Lactate content. Mean ± SEM, *N* = 8. Equal letters represent significant differences.

Naïve WAR did not differ from naïve Wistar rats in mitochondrial respiratory parameters in cortex biopsies (Figure 1B), despite elevated mitochondrial density (Figure 4A). Diversely, chronic acoustic stimuli induced an increase in oxygen consumption in cortex of the Wistar group in NADH-Linked, OXPHOS, and LEAK states (Figure 1B), and elevated uncoupling degree (Figure 1D). In accordance with the hippocampus, WAR and audiogenic kindled groups presented a more oxidized state–reduced GSH/GSSG (Figure 4B) and elevated oxidized proteins (Figure 4E) when compared to Wistar. Audiogenic kindling in WAR and chronic stimulation in Wistar groups resulted in a strong reduction in catalase activity (Figure 4C). No changes were observed in SOD activity (Figure 4D) and lactate content (Figure 4F) in the cortex of all groups.

Together, these results show that naïve WARs have an elevated mitochondrial metabolism/content and oxidative stress in hippocampus and cortex. In order to verify the relationship between mitochondrial energy metabolism and oxidative stress and seizure severity, we have treated WAR with 2,4-dinitrophenol (DNP) or N-acetylcysteine (NAC), two effective *in vivo* antioxidant strategies: the first acts by decreasing mitochondrial reactive oxygen species release by inducing mild mitochondrial uncoupling (13); the second is able to increase redox potential by elevating glutathione synthesis (18).

### Effects of *in vivo* Treatment With NAC or DNP on Seizure Severity Index (cSI) of WAR

A sound stimulus was performed before and after NAC or DNP treatments and cSI was measured. Afterwards, animals were divided into three groups, balancing the same seizure behaviors (cSI = 4.2). After the treatment period, the cSI were 7.25 ± 0.16 for WAR group, 7.00 ± 0.21 for WAR-DNP group, and 5.80 ± 0.57 for WAR-NAC group (*p* < 0.05 vs. WAR). Only *in vivo* treatment with NAC reduces cSI in WAR.

### Effects of *in vivo* Treatment With NAC or DNP on Mitochondrial Metabolism and Redox State in Hippocampus and Cortex of WAR

**Figure 5:**
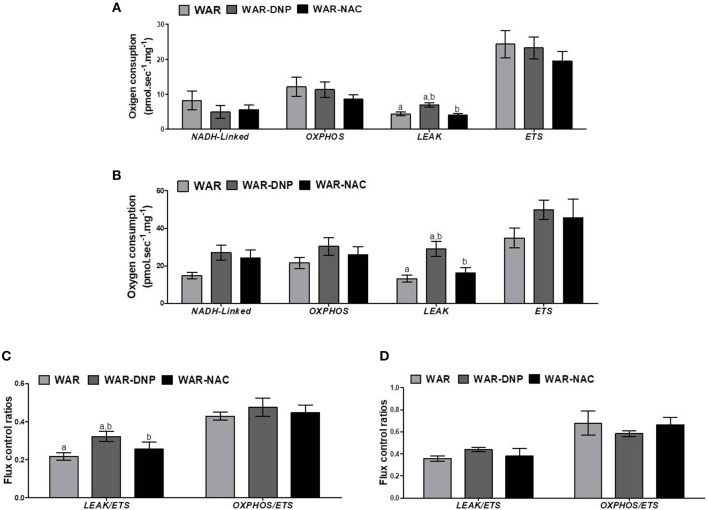
Mitochondrial respiration in brain biopsies from WAR, and WAR treated with DNP or NAC. Oxygen consumption in states of NADH-Linked, OXPHOS, LEAK, NON-COUPLED (ETS) in **(A)** hippocampus, or **(B)** cortex. Flux control ratios (FCRs) in **(C)** hippocampus or **(D)** cortex. Mean ± SEM, *N* = 8. Equal letters represent significant differences.

**Figure 6:**
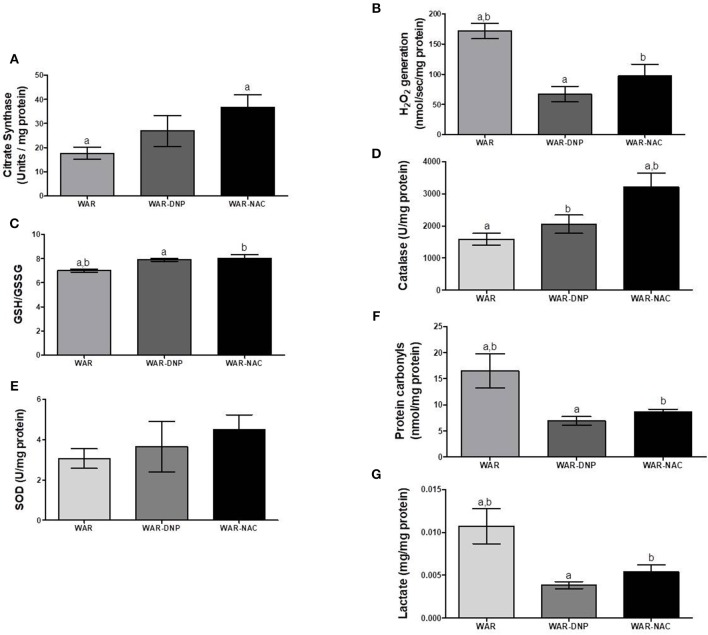
Mitochondrial content and redox state in hippocampus biopsies from WAR, and WAR treated with DNP or NAC. **(A)** Citrate synthase content, **(B)** H_2_O_2_ release, **(C)** GSH/GSSG ratio, **(D)** Catalase activity, **(E)** Superoxide Dismutase (SOD) activity, **(F)** Protein carbonyl content, and **(G)** lactate content. Mean ± SEM, *N* = 8. Equal letters represent significant differences.

**Figure 7:**
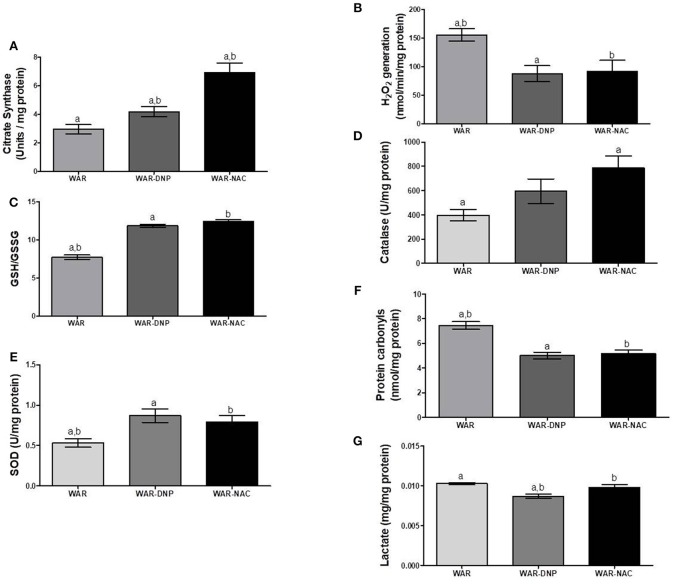
Mitochondrial content and redox state in cortex from WAR, and WAR treated with DNP or NAC. **(A)** Citrate synthase content, **(B)** H_2_O_2_ release, **(C)** GSH/GSSG ratio, **(D)** Catalase activity, **(E)** Superoxide Dismutase (SOD) activity, **(F)** Protein carbonyl content, and **(G)** lactate content. Mean ± SEM, *N* = 8. Equal letters represent significant differences.

As expected, treatment with DNP increased oxygen consumption in LEAK state in the hippocampus and cortex (Figures 5A,B), whilst uncoupling degree elevation was seen only in hippocampus (Figures 5C,D), known uncoupling effects of DNP, without significant change in mitochondrial density (Figure 6A) in biopsies of hippocampus (WAR-DNP). Contrarily, treatment with NAC (WAR-NAC) did not change any respiratory rates (Figures 5A–C), in spite of increased citrate synthase content (Figures 6A, 7A). Both treatments corrected the elevated lactate levels on hippocampus but only DNP promoted a slight decrease on cortex (Figures 6G, 7G). Oxidized state of WAR was diminished, evidenced by increased GSH/GSSG (Figures 6C, 7C), decreased protein carbonyl content (Figures 6F, 7F) and H_2_O_2_ release (Figures 6B, 7B). Catalase activity was strongly induced by NAC and DNP treatment in the hippocampus (Figure 6D), while in cortex, only NAC treatment had this effect (Figure 7D). Significant changes were observed in SOD activity only in the cortex for both treatments (Figures 6E, 7E).

Since *in vivo* DNP treatment is able to reduce mitochondrial-derived ROS, our results demonstrate that oxidative stress is associated with higher mitochondrial activity in hippocampus and cortex of WAR. Both *in vivo* antioxidant strategies resulted in reductions on oxidative stress markers, without diminishing mitochondrial content. However, only NAC treatment was able to reduce cSI in WAR. Our last question was regarding the ability of *in vivo* NAC treatment to promote some additional changes in energetic metabolism in WAR besides lowering oxidative stress. Hence, we investigated the expression of genes related to carbohydrate, lactate and amino acid metabolisms in Wistar and WAR, submitted to *in vivo* DNP or NAC treatments or not (naïve).

### Expression of Genes Related to Carbohydrate, Lactate, and Amino Acid Metabolisms in Hippocampus of Wistar and WAR Naïve or Submitted to *in vivo* DNP or NAC Treatments

**Figure 8:**
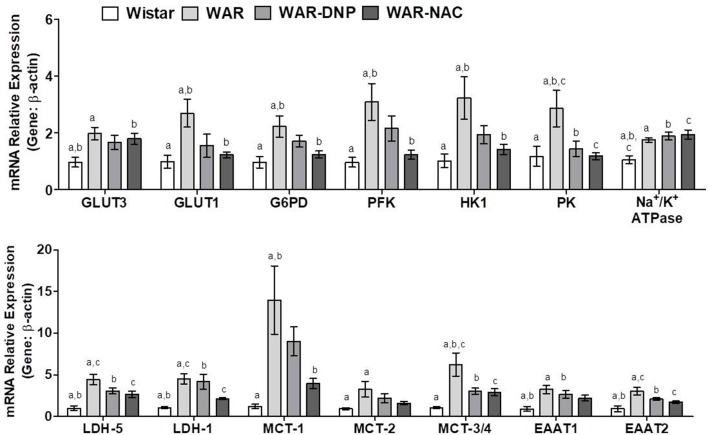
mRNA expression of genes related to energetic metabolism in hippocampus from Wistar, WAR, and WAR treated with DNP, or NAC. GLUT3 and GLUT1, Glucose Transporters; G6PD, Glucose-6-Phosphate dehydrogenase; HK1, Hexokinase 1; PK, Pyruvate kinase; PFK, Phosphofructokinase; EAAT1 and EAAT, transporters of excitatory amino acids; Na^+^/K^+^ ATPase, MCT1, MCT-2, and MCT-3/4, monocarboxylate transporters; and LDH-1 and LDH-5, lactate dehydrogenases. Mean ± SEM, *N* = 8. Equal letters represent significant differences.

Compared to Wistar rats, hippocampus of WAR presented elevated mRNA expression of glucose transporters isoform-3 (GLUT3, predominant in neurons), isoform-1 (GLUT1, predominant in astrocytes), and glycolytic enzymes Glucose-6-Phosphate Dehydrogenase (G6PD), Phosphofructokinase (PFK), Hexokinase 1 (HK1), and Pyruvate Kinase (PK) (Figure 8). DNP or NAC treatments reversed the high expression of all these enzymes, except to GLUT3. In addition, hippocampus of WAR showed elevated mRNA expression of genes involved in lactate metabolism, such as Lactate Dehydrogenase isoform-1 (LDH-1, predominant in neurons) and isoform-5 (LDH-5, predominant in astrocytes) and lactate transporters Monocarboxylate Transporters isoform-1 (MCT-1, predominant in astrocytes), isoform-2 (MCT-2, predominant in neurons) and isoforms-3 or−4 (MCT-3/4). NAC treatment was able to reverse all these elevations, whilst DNP treatment only reversed MCT-3/4. Hippocampus of WAR also presented augmented mRNA expression of genes involved in transport of excitatory amino acids isoform-1 (EAAT1), isoform-2 (EAAT2), and Na^+^/K^+^ ATPase. Among these, only EAAT2 gene expression was reversed by NAC treatment.

Together, these results suggest an accentuated transport and metabolism of glucose and lactate in hippocampus of WAR. Due to the near absence of mitochondria, accelerated glucose uptake followed by glycolysis in astrocytes culminates in higher lactate production, which is exported to neurons. The latter, in turn, convert lactate into pyruvate, in addition to the pyruvate provided by its own glycolysis, to fuel the high mitochondrial metabolism with acetyl-CoA. Higher mitochondrial content and phosphorylation capacity improves the supply of ATP to Na^+^/K^+^ ATPase pumps and, consequently, increase ROS production. NAC treatment, in WAR, reversed oxidative stress, and unlike DNP, reduced gene expression of almost all enzymes involved in glucose uptake and metabolism in neurons and astrocytes (Figure 8). Since EAATs limit glutamatergic signaling and maintain extracellular glutamate concentrations below neurotoxic levels by removing this neurotransmitter of the synaptic cleft, as they transport the glutamate back to the original neuron and/or into adjacent astrocytes, the downregulation of EEAT2 by NAC may reflect a decrease of glutamate concentration released by seizures. When seizures are present—or other excitotoxic events—extracellular glutamate levels increase exponentially, when compared to a normal neurotransmission, and contribute to the reactive astrocytosis associated with epileptogenesis and other neurotoxic events (38).

### Expression of Genes Related to Carbohydrate, Lactate, and Amino Acid Metabolisms in Cortex of Wistar and WAR Naïve or Submitted to *in vivo* DNP or NAC Treatments

**Figure 9:**
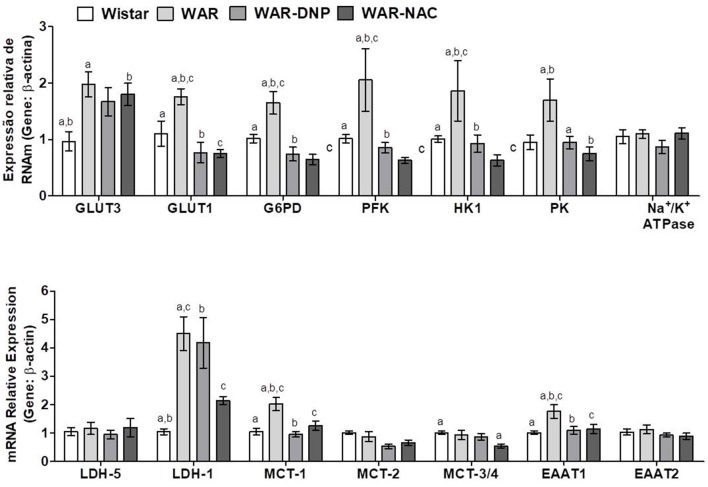
mRNA expression of genes related to energetic metabolism in cortex from Wistar, WAR, and WAR treated with DNP, or NAC. GLUT3 and GLUT1, Glucose Transporters; G6PD, Glucose-6-Phosphate dehydrogenase; HK1, Hexokinase 1; PK, Pyruvate kinase; PFK, Phosphofructokinase; EAAT1 and EAAT, transporters of excitatory amino acids; MCT1, MCT-2, and MCT-3/4, monocarboxylate transporters; and LDH-1 and LDH-5, lactate dehydrogenases. Mean ± SEM, *N* = 8. Equal letters represent significant differences.

As found in hippocampus, cortex of WAR presented elevated mRNA expression of GLUT3, GLUT1, G6PD, PFK, HK1, PK, and EAAT1, which were reversed by DNP or NAC treatments, except for GLUT3 (Figure 9). Additionally, cortex of WAR showed elevated expression of LDH-1 and MCT-1, being the latter reversed by both DNP or NAC treatments, while LDH-1 was only reversed by NAC. No changes were found in the gene expression of LDH-5, MCT-2, MCT3/4, or Na^+^/K^+^ ATPase. Contrasting with the hippocampus, these results suggest an accentuated transport and metabolism of glucose, while a partial improvement in lactate metabolism in the cortex of WAR, which are reversed by both *in vivo* DNP or NAC treatments.

## Discussion

Our results revealed that in naïve (non-stimulated) WAR, the cerebral cortex and hippocampus have a highly efficient powerhouse, characterized by elevated mitochondrial density and/or activity, which leads to increased oxidized state and oxidative damage (39). Oxidative damage is a contributing factor to epileptogenesis, and regulation of oxidative stress with antioxidant therapies has been used as a mean of attenuating this pathology and seizure initiation [for a review (40)]. However, oxidative stress does not have the same pattern in all seizure models. In the pilocarpine-induced model of Status Epilepticus (SE), a reduction of GSH is observed (41, 42), leading to excessive lipid peroxidation in the hippocampus and cortex (43); in kainic acid-induced seizures, there is an overproduction of ROS associated with mitochondrial dysfunction and calcium overload mediated by excessive glutamate receptor activation, leading to the damage of cell structures and alterations including hippocampal lipids and proteins (44). In audiogenically susceptible strains such as GEPRs (acute seizures), a breakdown in hippocampal glutathione peroxidase (GPx) activity has been described, with a decrease in GSH/GSSG ratio (40, 45), while in the whole brain of the Krushinsky-Molodkina (KMs) rat an increased ROS generation by mitochondrial complex I dysfunction was observed (46).

Although we have observed several profound alterations in mitochondrial physiology and biochemistry in naïve WARs, there is no effect of audiogenic kindling in WAR. Curiously, chronic auditory stimuli in Wistar rats induced alterations observed in naïve WAR, such as increased mitochondrial respiration at OXPHOS state and oxidative state. In addition, chronic auditory stimuli induced respiration at LEAK states in Wistar rats, an effect which increases energy expenditure and decreases mitochondrial ROS generation (47). To our knowledge, these effects have not been observed yet. We believe that chronic stimulation in Wistar acted as an environmental stress to these animals, since high levels of circulating glucocorticoid hormone may be important mediators for elevating mitochondrial biogenesis upon stress, as described in skeletal muscle (48). In cortical neurons, mitochondrial function is modulated depending on corticosterone doses (49). There are still questions regarding the inability of audiogenic stimulation to change the already altered mitochondrial status of susceptible, but naïve WAR (which, when submitted to audiogenic kindling, displays brainstem and limbic seizures). Is there any compensatory mechanism associated to this kind of refractoriness or protection? Or is WAR already in a sort of ceiling-effect state?

The *in vivo* antioxidant strategies using DNP and NAC were effective and demonstrated that, additionally to oxidative stress, other biochemical components in the hippocampus and cortex of naïve WAR can be involved in epilepsy and seizure severity. In a previous study, the concentration of GLUT4 in the gastrocnemius muscle of WAR was found to be 1.6-fold higher than that observed in Wistar rats (50). NAC treatment also reverted the elevated gene expression of almost all enzymes involved in glucose and lactate uptake and metabolism in neurons and astrocytes, mainly in the hippocampus of WAR. When down regulating these exacerbated metabolic routes, less ATP can be available to fuel Na^+^/K^+^ ATPases, a key enzyme in brain excitability, which is sensitive to the redox state (51). The higher mRNA expression of Na^+^/K^+^ ATPase found in our study is consistent with higher Na^+^/K^+^ ATPase activity observed in forebrain samples of naïve WAR (50).

The metabolic effects of NAC observed are very similar to those obtained using ketogenic diet (low carbohydrates, high lipids and proteins) (52–55). Reductions in the progression of kindling in a rat model of temporal lobe epilepsy (a drug-resistant epilepsy model that sometimes responds to dietary manipulation such as the ketogenic diet) were achieved using the glycolytic inhibitor 2-deoxy-D-glucose (56). The anti-glycolytic effects of NAC were firstly described in skeletal muscle, where it reduced GLUT4 content, PFK activity and consequently lactate production (57, 58). Here, we suggest that NAC modulates neuronal excitability and the appearance or propagation of seizures through its antioxidant capacity and ability to reduce glycolytic/lactic metabolism in WAR. In a pilocarpine-induced SE model, NAC was found to increase the interval between seizures, exerting a dose-dependent anticonvulsant effect on acute and chronic use (59).

In a previous study of our group, we found the same mitochondrial and oxidative profile in the liver, soleus muscle and cardiac tissue of WAR (60). In these organs, the changes were associated with higher expression of peroxisome proliferator-activated receptor gamma coactivator 1-alpha (PGC1α) and mammalian target of rapamycin (mTOR). In the brain, excessive activation of mTOR signaling is often linked to the development of epilepsy, and mTOR inhibitors have consistent protective effects in various epilepsy animal models and epileptic patients (61, 62). In this regard, the mitochondrial changes found in the present study could be associated with the activation of the mTOR pathway in cortex and hippocampus of naïve WAR.

## Conclusion

**Figure 10:**
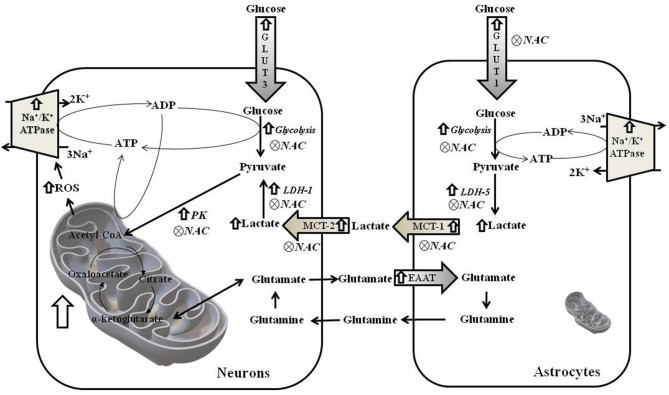
Schematic figure of metabolism of neurons and astrocytes from hippocampus of WAR. ROS, reactive oxygen species; GLUT3 and GLUT1, Glucose Transporters; EAAT1 and EAAT, transporters of excitatory amino acids; PK, Pyruvate kinase; MCT1 and MCT-2, monocarboxylate transporters; and LDH-1 and LDH-5, lactate dehydrogenases.

In WAR, the cerebral cortex and hippocampus display elevated mitochondrial density and/or activity associated with oxidative damage, upregulated genes of glucose and lactate metabolisms, and overexpression of Na^+^/K^+^ ATPase (Figure 10). Only *in vivo* antioxidant therapy with NAC was able to downregulate genes involved in glucose/lactate metabolisms and effectively reduce seizure severity.

## Data Availability

The datasets generated for this study are available on request to the corresponding author.

## Ethics Statement

The animal study was reviewed and approved by Ethics Committee on the use of animals at USP (CEUA, 011/2015).

## Author Contributions

CD conducted all the experiments. JO provided rats from the breeding colony. GF made primer sequences and corrected the English version. GF and JS conducted the experiments of respiration. JO and CD conducted the experiments regarding audiogenic kindling. RS-J and AC conducted the image experiments. CD, NG-C, and LA conceived the idea of the experimental design and analyzed all the results. CD, RS-J and NG-C wrote part of the paper. LA wrote the paper. All authors have approved the final version of the manuscript and agree to be accountable for all aspects of the work.

### Conflict of Interest Statement

The authors declare that the research was conducted in the absence of any commercial or financial relationships that could be construed as a potential conflict of interest.
